# Differential Protective Actions of Dulaglutide and Dexamethasone in Methotrexate Induced Pulmonary Fibrosis: Relationship with AMPK and Beclin-1 Levels

**DOI:** 10.3390/biomedicines14081714

**Published:** 2026-07-30

**Authors:** Omar W. Maher, Norhan M. El-Sayed, El-Sayed E. El-Awady, Naglaa F. El-Orabi, Asmaa Radwan

**Affiliations:** 1 Department of Pharmacology and Toxicology, Faculty of Pharmacy, Tobruk University, Tobruk, Libya; omarpharm638@gmail.com; 2 Department of Pharmacology and Toxicology, Faculty of Pharmacy, Suez Canal University, Ismailia 41522, Egypt; sayed.alawadi@pharm.suez.edu.eg (E.-S.E.E.-A.); naglaa_elorabi@pharm.suez.edu.eg (N.F.E.-O.)

**Keywords:** methotrexate, dulaglutide, pulmonary fibrosis, oxidative stress, inflammation

## Abstract

**Background and Objectives**: Pulmonary fibrosis (PF) is a life-threatening respiratory disorder involving complex pathophysiological mechanisms such as inflammation, collagen deposition, and epithelial cell injury. Methotrexate (MTX), a chemotherapeutic and immunosuppressive agent, is frequently associated with pulmonary toxicity, representing a significant adverse effect with unpredictable outcomes. Thus, the current study investigates the possible protective effect of dulaglutide (DUL) against MTX-induced lung injury. **Methods**: Sixty male Wistar rats were allocated into six groups: group 1 served as a control group, group 2 received MTX (14 mg/kg/week, p.o) for 2 weeks, group 3 was treated with MTX treatment (14 mg/kg/week, p.o) prior to dexamethasone (DEXA) (0.5 mg/kg/week i.p), Groups 4–5 were treated concurrently with MTX (14 mg/kg/week, p.o) and DUL at doses of 0.05 and 0.1 mg/kg/week, s.c. and Group 6 received DUL (0.1 mg/kg/week, s.c.) only. Animals were sacrificed on day 15 for histopathological and biochemical assessments. **Results**: Our data demonstrated that MTX markedly increased some oxidative stress markers, inflammatory biomarkers and fibrotic and apoptotic indicators, alongside a significant decrease in Beclin-1 and Adenosine Monophosphate Activated Protein Kinase (AMPK) levels. DUL administration significantly ameliorated these alternations in a dose-dependent manner, with histopathological findings corroborating biochemical data through Hematoxylin & Eosin (H&E) and Mason’s Trichrome (MTC) staining. **Conclusions**: DUL confers significant protection against MTX-induced pulmonary fibrosis, likely through its antioxidant, anti-inflammatory, and anti-apoptotic properties, which are associated with the restoration of total AMPK and Beclin-1 levels. This highlights its therapeutic potential in preventing drug-induced lung toxicity.

## 1. Introduction

Pulmonary fibrosis (PF) is a major worldwide health issue that is characterized by progressive, irreversible scarring of the lung tissue, leading to severe respiratory impairment and high mortality rates [1]. The most prevalent type of pulmonary fibrosis is idiopathic pulmonary fibrosis (IPF) [2]. During 2020 and 2022, a substantial number of deaths were attributed to IPF, with over 67,000 decedents in the United States alone, highlighting its profound public health impact. Reports indicate an annual age-adjusted IPF mortality rate of 7.1 per 100,000 people with higher rates observed among older adults [3]. The progressive nature of the disease, coupled with limited effective treatments, contributes to a poor prognosis and a considerable burden on healthcare systems and patient quality of life [4]. While various factors contribute to the development of pulmonary fibrosis, drug-induced forms, such as methotrexate-induced pulmonary fibrosis (MTX-PF), are also of clinical concern. Methotrexate, a widely used antimetabolite, can lead to pulmonary toxicity, with reported incidences ranging from 0.1 to 1% for pneumonitis—a form of lung toxicity that can precede fibrosis in patients on low-dose therapy. Studies suggest that between 1% and 2% of individuals with rheumatoid arthritis experience severe pulmonary side effects with methotrexate [5]. Although high-quality evidence directly linking methotrexate to chronic interstitial lung fibrosis in humans remains limited, MTX is identified as the second most common drug involved in drug-induced pulmonary fibrosis in a national database analysis [6].

Methotrexate, an antagonist to folic acid, is commonly used to treat various cancers—including acute lymphoblastic leukemia, sarcoma, lung and bladder cancer—as well as inflammatory diseases, including multiple sclerosis and rheumatoid arthritis [7]. Pulmonary toxicity, especially fibrosis, along with toxic effects on the brain, kidney, liver are among drug’s negative consequences [8,9].

The mechanisms underlying MTX-induced pulmonary fibrosis are complex. It is suggested that MTX triggers oxidative damage and inflammation, leading to lung injury [10]. In this process, increased levels of oxidative stress markers like malondialdehyde (MDA) are observed, while antioxidant defenses such as glutathione (GSH) and antioxidant enzyme activities decrease [11]. In addition, MTX induces apoptosis, evidenced by elevated Bax and reduced Bcl-2 expression levels [12]. Bax promotes apoptosis by facilitating the release of mitochondrial cytochrome c, which in turn activates caspase-3 [13]. Interestingly, Bax also plays a role in inflammation and oxidative stress responses, further contributing to cell death [14].

Beyond inflammation, emerging research highlights the role of autophagy—a cellular self-digestion process—in the development of MTX-PF. While autophagy generally serves a cytoprotective role by removing damaged organelles and proteins, its dysregulation can contribute to disease pathology [15]. While cytoprotective autophagy serves to clear damaged organelles and alleviate cellular stress, its suppression is increasingly implicated in drug-induced pulmonary injury. Recent evidence indicates that MTX exposure impairs cellular defense mechanisms by downregulating key autophagic markers, such as Beclin-1, thereby accelerating cell death and fibrogenesis in the lung [16,17].

For instance, a study has shown that MTX-induced lung toxicity involves increased oxidative stress and inflammation, and that modulation of autophagy pathways influences fibrosis progression [18]. Understanding the intricate balance and interplay between inflammatory responses and autophagic pathways is crucial for elucidating MTX-PF pathogenesis and identifying potential therapeutic targets.

In rheumatoid arthritis, methotrexate has been shown to induce autophagy via increased Beclin-1 expression, bypassing the Akt/mTOR pathway—a key regulator of autophagy [19]. In both cellular signaling and damage, autophagy has become a crucial mediator of pathogenic responses and is linked to reactive oxygen species (ROS) [20].

Additionally, abnormal myofibroblast activation—a key driver of extracellular matrix increased stiffness—is indicated by increased Alpha-smooth muscle actin (α-SMA) levels, a marker protein used to assess fibroblasts activation in various organs and tissues, including the lung [21]. Enhanced collagen secretion for post-injury healing and epithelial–mesenchymal transition (EMT) further contribute to fibrosis [22]. Pulmonary damage also causes an imbalance between antifibrotic molecules—such as prostaglandin E2 and interferon-γ (IF-γ)—and profibrotic factors such as transforming growth factor beta 1 (TGF-β1) and thrombin. This imbalance promotes increased extracellular matrix (ECM) synthesis and a shift in apoptotic cell behavior and proliferation [23]. Notably, elevated levels of growth factors and pro-inflammatory cytokines like tumor necrosis factor-alpha (TNF-α), interleukin-4 (IL-4), and TGF-β increase the fibroblasts proliferation and production of extracellular matrix proteins like fibrin [24].

Dulaglutide is a glucagon-like peptide-1 (GLP-1) analog, derived from the glucagon gene in small intestine L cells following food intake. It enhances glucose-dependent insulin secretion by interacting with specific GLP-1 receptors in the pancreas [25]. Beyond its glycemic effects, GLP-1 exhibits anti-inflammatory, antioxidative, neuroprotective, and vasoprotective activities across various tissues and cell types, including the lungs, kidney, heart, endothelial cells and pancreatic beta cells. Emerging evidence indicates that GLP-1 signaling can directly arrest fibrotic progression by upregulating cellular energy sensors like AMPK, restoring cytoprotective autophagy via Beclin-1 activation, and downregulating pro-fibrotic mediators like TGF-β1 and α-SMA. Consequently, dulaglutide was selected as a novel therapeutic candidate to target these key pathological nodes in MTX-induced lung injury [26,27,28,29].

In the present study, a methotrexate (MTX)-induced lung fibrosis model was used to examine the potential therapeutic effects of dulaglutide (DUL). Dexamethasone (DEXA) is commonly used to alleviate the severity of fibrosis and alveolitis in PF, by rapidly suppressing the upstream inflammatory cytokine cascade (such as TNF-α and IL-4) and preventing alveolar epithelial cell apoptosis [30]. Because corticosteroids are the clinical standard of care for acute drug-induced pneumonitis and early-stage pulmonary injury, DEXA serves as a mechanistically relevant benchmark to evaluate the comparative anti-inflammatory, anti-apoptotic, and anti-fibrotic efficacy of dulaglutide.

## 2. Materials and Methods

### 2.1. Animals

Adult male Wistar rats, weighing between 170 and 200 g, were obtained from the National Center of Research (Cairo, Egypt). Prior to the start of the experiments, the animals were acclimatized in the animal house for two weeks. They were kept in standard laboratory conditions, including a 12-h light/dark cycle, 60% humidity, and unrestricted access to food and water. The experiments were conducted in accordance with the ethical guidelines for the care and use of laboratory animals. The study protocol was approved by the Research Ethics Committee of the Faculty of Pharmacy, Suez Canal University (Approval No. 202212A1).

### 2.2. Drugs

Methotrexate (MTX) was provided by Hikma Specialized Pharmaceutics, Cairo, Egypt. Dulaglutide (DUL) was generously supplied by Eli Lilly and Company, Indianapolis, IN, USA. Dexamethasone (DEXA) was given by Medical Union Pharmaceutics, Cairo, Egypt.

### 2.3. Experimental Design

**Figure 1:**
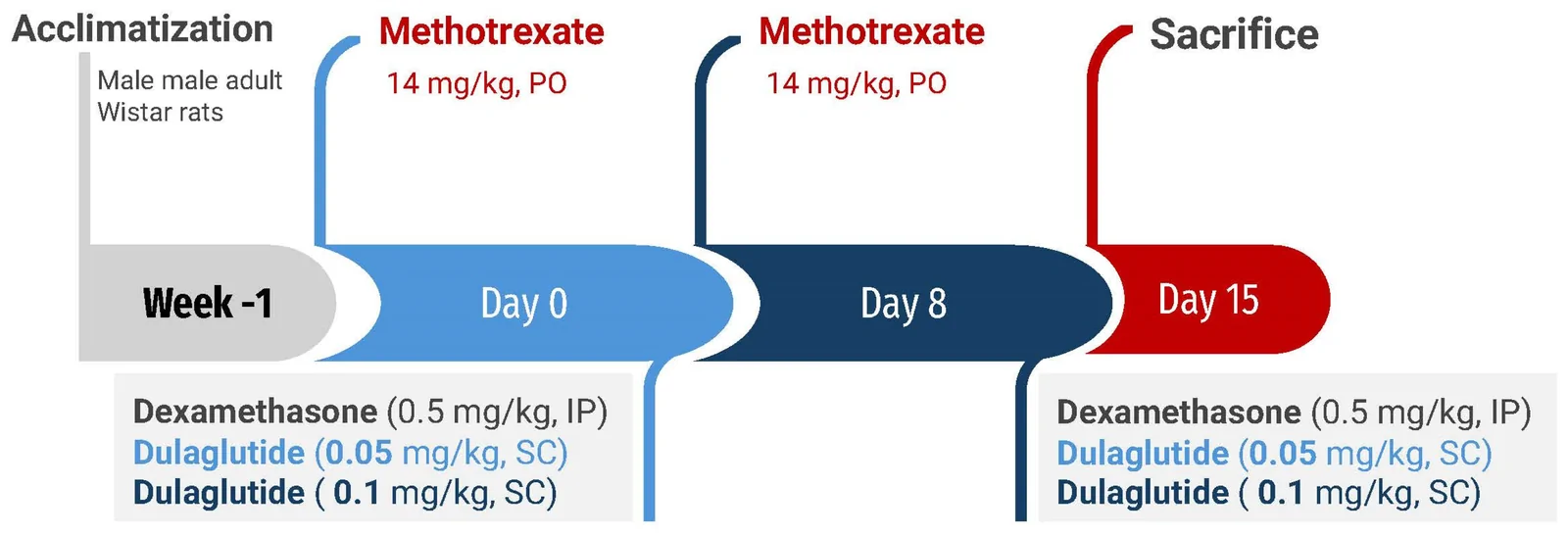
The Experimental Timeline.

Sixty male adult Wister rats were randomly allocated into six experimental groups (*n* = 10 per group). Group 1 served as the control and received saline orally. Group 2 (fibrotic control) was administered methotrexate (MTX) at a dose of 14 mg/kg/week orally for two weeks. This MTX dosing regimen was selected based on established literature showing it reliably induces acute pulmonary injury and early-stage fibrosis within a brief 2-week period. Group 3 received MTX (14 mg/kg/week, p.o.) concomitantly with dexamethasone (DEXA, 0.5 mg/kg/week, i.p.) for two weeks. The weekly administration schedule for dexamethasone (0.5 mg/kg/week, i.p.) was chosen to mirror the once-weekly dosing regimen of MTX and to prevent severe corticosteroid-induced systemic toxicity or metabolic complications associated with daily high-dose administration. Groups 4 and 5 were treated concurrently with MTX (14 mg/kg/week, p.o.) and dulaglutide (DUL) at doses of 0.05 mg/kg/week and 0.1 mg/kg/week, respectively, administered subcutaneously. The dulaglutide doses (0.05 and 0.1 mg/kg/week, s.c.) were chosen based on previous rodent studies demonstrating their anti-inflammatory and tissue-protective efficacy without causing hypoglycemia in non-diabetic animals. Group 6 received DUL alone at 0.1 mg/kg/week, s.c., for two weeks. The experimental timeline for this study is shown in Figure 1.

### 2.4. Sample Preparation

At the end of the experiment, rats were anesthetized with thiopental sodium anesthesia (50 mg/kg IP). Following sacrifice, the lung tissues were excised and thoroughly rinsed with saline. The right lung’s lobes were immediately snap-frozen at −80 °C for subsequent ELISA and quantitative RT-PCR analyses. The left lung lobes were fixed in 10% neutral-buffered formalin, embedded in paraffin, and sectioned into 5 µm slices using a rotary microtome for histopathological and immunohistochemical examinations.

### 2.5. Histopathological and Immunohistochemical Examination of the Lung Tissues

For the histopathology, Lung sections from different experimental groups were mounted on glass slides, deparaffinized, and stained with hematoxylin and eosin (H&E) for general histological evaluation and Masson’s Trichrome (MTC) stain for assessment of collagen deposition. The stained sections were examined under a light microscope.

Immunohistochemical staining for BAX was performed on formalin-fixed, paraffin-embedded lung section (3 μm) from all experimental groups using a rabbit monoclonal anti-BAX antibody (E63, ab 32503, Abcam, Cambridge, UK) at a dilution of 1:200. Antigen retrieval was performed by heating in citrate buffer (pH 6.0) for 20 min in a microwave oven (Microwave Research & Applications, Laurel, MD, USA), followed by blocking of endogenous peroxidase activity with 3% hydrogen peroxide for 10 min at room temperature. Sections were then incubated with the secondary antibody incubation system (EnVision + HRP anti-rabbit polymer detection system, K4003, Dako, Glostrup, Denmark) and visualized using 3,3-diaminobenzidine (DAB) chromogen. Hematoxylin was applied as a counterstain, while negative controls were prepared by replacing the primary antibody with phosphate-buffered saline.

Digital photographs of Masson’s Trichrome (MTC) and BAX-stained lung sections were captured at 40× magnification. We quantified the collagen deposition area (MTC area %) and pro-apoptotic marker expression (BAX area %) using ImageJ software (version 1.54). All histological slide evaluations, immunohistochemical scoring, and quantitative morphometric analyses (ImageJ area percentage measurements) were performed by a pathologist who was blinded to the treatment group assignments.

### 2.6. Determination of Fibrotic Markers Using Real-Time PCR Analysis

The expression levels of collagen-1, α-SMA, MMP-9, CTGF, SMAD3, and SMAD7 genes in lung homogenate were quantified using a quantitative real-time PCR. Total RNA from lung tissue was extracted using the miRNeasy Mini Kit Isolation kit (Qiagen, Hilden, Germany) in accordance with the manufacturer’s instructions. Spectrophotometry (NanoDrop Tech., Wilmington, DE, USA) was used to measure the extracted RNA’s concentration and purity. Gene expression analysis was performed using GoTaq^®^ 1-Step RT-qPCR System (Promega, Madison, WI, USA) and β-actin as a housekeeping control. Each reaction mixture contained 10 µL of GoTaqR qPCR master mix, 0.4 µL of GoScript™ reverse transcriptase mix, 0.31 µL of CXR reference dye, 3.29 µL of nuclease-free water, 4 µL of RNA template, and 1 µL of each forward and reverse primers. Amplification was carried out on StepOne real-time PCR System (Applied Biosystems, Waltham, MA, USA) under the following conditions: denaturation at 95 °C for 10 s, annealing at primer-specific temperature for 30 s, and extension at 72 °C for 30 for a total of 40 cycles. The reaction was run in duplicate without a control. The 2-ΔΔCT method was used to calculate the relative gene expression and expressed as fold change. Primer sequences, annealing temperatures, and accession numbers are listed in Table 1.

### 2.7. Biochemical Assays

Lung tissue homogenates were analyzed for oxidative stress, cytokine, apoptosis, protein kinase, and autophagy markers using commercially available enzyme-linked immunosorbent assay (ELISA) kits in accordance with the manufacturers’ protocols. Oxidative stress and antioxidant parameters included malondialdehyde (MDA; MyBioSource, Cat. No. MBS268427, San Diego, CA, USA), reduced glutathione (GSH; Shang Hai Blue Gene Biotech, Cat. No. E02G0367, Shanghai, China), catalase (CAT; MyBioSource, Cat. No. MBS036924, San Diego, CA, USA), superoxide dismutase (SOD; MyBioSource, Cat. No. MBS006963, San Diego, CA, USA), and nuclear factor erythroid 2–related factor 2 (Nrf2; MyBioSource, Cat. No. MBS765897, San Diego, CA, USA). Proinflammatory cytokines were quantified using TNF-α (MyBioSource, Cat. No. MBS2507393, San Diego, CA, USA) and IL-4 (Elabscience, Cat. No. E-EL-R0014, Houston, TX, USA) kits. Apoptotic markers were measured using TGF-β1 (MyBioSource, Cat. No. MBS011634, San Diego, CA, USA) and caspase-3 (MyBioSource, Cat. No. MBS261814, San Diego, CA, USA) kits. Protein kinase activity was determined by measuring AMPK levels (MyBioSource, Cat. No. MBS752046, San Diego, CA, USA). Autophagy was assessed by quantifying Beclin-1 levels (MyBioSource, Cat. No. MBS2706719, San Diego, CA, USA).

### 2.8. Statistical Analysis

Data are presented as mean ± standard error of mean (SEM). Statistical analyses were performed using GraphPad Prism version 10 (GraphPad Software Inc., La Jolla, CA, USA). One-way analysis of variance (ANOVA) followed by Tukey–Kramer multiple comparison post hoc test was applied to evaluate differences between groups. A *p*-value of less than 0.05 was considered statistically significant.

## 3. Results

### 3.1. Histopathological Evaluation of Dulaglutide’s Effects Against Methotrexate-Induced Pulmonary Fibrosis in Rats

**Figure 2:**
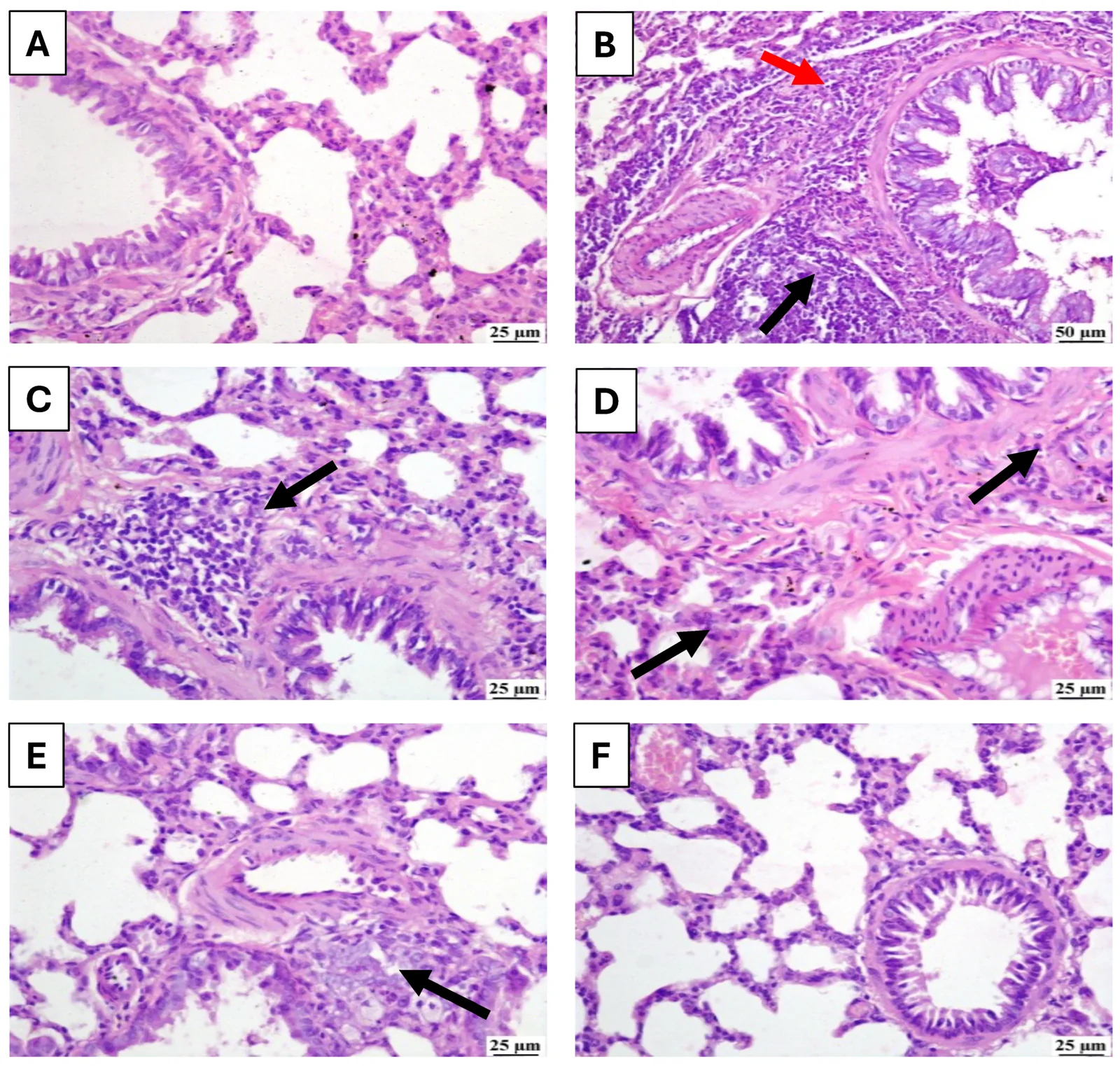
Photomicrographs of lung sections (H&E; 40×) showing the effect of Dulaglutide on methotrexate (MTX)–induced pulmonary fibrosis. (**A**): control group showing normal bronchiole and alveoli, (**B**): MTX group showing peribronchiolar fibroplasia (red arrow) with mononuclear inflammatory cells infiltrations (black arrow) (**C**): MTX + DEXA group showing mild focal mononuclear inflammatory cells infiltration (arrow), (**D**): MTX + DUL 0.05 group showing moderate perivascular and peribronchiolar fibroplasia (arrow), (**E**): MTX + DUL 0.1 group showing mild perivascular fibroplasia (arrow), (**F**): DUL 0.1 group showing normal lung structure including bronchioles and alveoli.

Histological evaluation of lung tissue sections stained with Hematoxylin and Eosin (H&E) revealed distinct pathological alterations among the experimental groups (Figure 2). The control group (A) displayed normal lung architecture with intact bronchioles and alveoli, indicative of healthy pulmonary tissue. In contrast, the MTX-treated group (B) showed severe pathological changes, including pronounced peribronchiolar fibroplasia and dense infiltration of mononuclear inflammatory cells, confirming the development of fibrotic and inflammatory lung injury. Treatment with dexamethasone (MTX + DEXA group, C) resulted in a noticeable reduction in tissue damage, with only mild focal infiltration of inflammatory cells, suggesting partial protective effects. Similarly, dulaglutide at 0.05 mg/kg (MTX + DUL 0.05 group, D) showed moderate perivascular and peribronchiolar fibroplasia, comparable to the DEXA group. However, dulaglutide at 0.1 mg/kg (MTX + DUL 0.1 group, E) demonstrated superior histological improvement, with only mild perivascular fibroplasia, indicating a dose-dependent protective effect that surpassed that of dexamethasone. The DUL 0.1 group (F), which received dulaglutide alone, exhibited normal lung histology, confirming the safety of DUL in the absence of MTX-induced injury.

**Figure 3:**
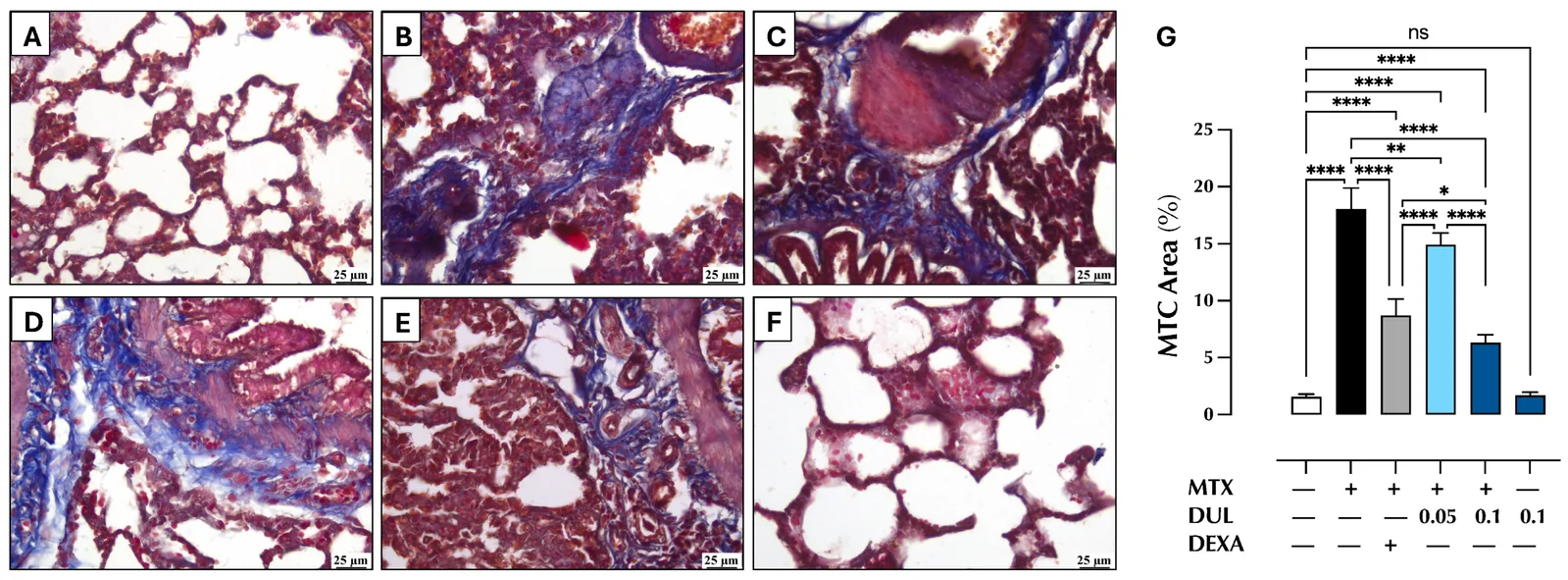
Photomicrographs of lung sections by Masson’s trichome (MTC; 40×) showing the effect of Dulaglutide on methotrexate (MTX)–induced pulmonary fibrosis. (**A**): control group showing normal lung structure, (**B**): MTX group showing abundant fibroplasia, (**C**): MTX + DEXA group showing moderate fibroplasia, (**D**): MTX + DUL 0.05 group showing moderate fibroplasia, (**E**): MTX + DUL 0.1 group showing lower fibroplasia, (**F**): DUL 0.1 group showing normal lung structure, (**G**): MTC area % in different groups. Statistical analysis was performed by one-way ANOVA followed by Tukey’s multiple comparisons test, *p*-values were coded as follows: ns, non-significant, * *p* < 0.05, ** *p* < 0.01, **** *p* < 0.0001.

Masson’s Trichrome (MTC) staining was used to assess collagen deposition and fibrotic changes (Figure 3). The control group (A) showed no evidence of collagen accumulation, while the MTX group (B) exhibited extensive fibroplasia with dense collagen deposition, confirming the induction of pulmonary fibrosis. The MTX + DEXA group (C) showed moderate collagen deposition, indicating partial attenuation of fibrosis. The MTX + DUL 0.05 group (D) also displayed moderate fibroplasia, similar to the DEXA group. Notably, the MTX + DUL 0.1 group (E) exhibited markedly reduced collagen accumulation, suggesting a stronger antifibrotic effect than DEXA. The DUL 0.1 group (F) showed normal lung structure with no signs of fibrosis, further supporting the safety profile of dulaglutide. Overall, dulaglutide demonstrated a dose-dependent protective effect against MTX-induced pulmonary damage, with the higher dose showing greater efficacy than dexamethasone in reducing both inflammation and fibrosis.

### 3.2. Effect of Dulaglutide on BAX Expression in Lung Tissue Following Methotrexate Treatment

**Figure 4:**
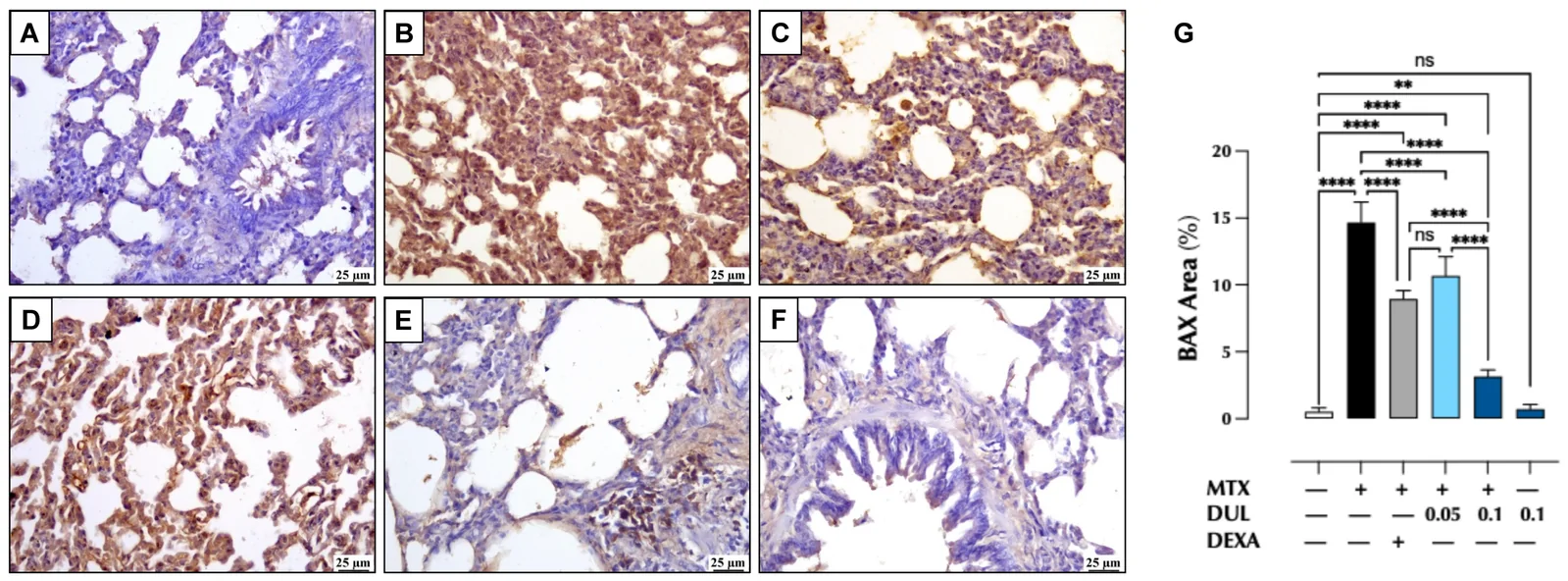
Effect of Dulaglutide on BAX Expression in Lung Tissue Following Methotrexate Treatment. (**A**): control group showing weak Bax expression, (**B**): MTX group showing higher Bax expression, (**C**): MTX + DEXA group showing moderate Bax expression, (**D**): MTX + DUL 0.05 group showing moderate Bax expression, (**E**): MTX + DUL 0.1 group showing lower Bax expression, (**F**): DUL 0.1 group showing weak Bax expression, (**G**): BAX area % in different groups. Statistical analysis was performed by one-way ANOVA followed by Tukey’s multiple comparisons test, *p*-values were coded as follows: ns, non-significant, ** *p* < 0.01, **** *p* < 0.0001.

Immunohistochemical analysis of lung tissue sections stained for BAX, a pro-apoptotic marker, revealed differential expression patterns across the experimental groups, as shown in Figure 4. The control group (A) exhibited weak BAX expression, consistent with normal apoptotic activity in healthy lung tissue. In contrast, the MTX-treated group (B) showed markedly increased BAX expression, indicating enhanced apoptotic activity associated with methotrexate-induced pulmonary damage. The MTX + DEXA group (C) demonstrated moderate BAX expression, suggesting partial suppression of apoptosis by dexamethasone. Similarly, the MTX + DUL 0.05 group (D) showed moderate BAX expression, comparable to the DEXA-treated group, indicating a protective effect of dulaglutide at the lower dose. Notably, the MTX + DUL 0.1 group (E) exhibited lower BAX expression than both the DEXA and DUL 0.05 groups, suggesting a superior anti-apoptotic effect of dulaglutide at the higher dose. The DUL 0.1 group (F), which received dulaglutide alone, showed weak BAX expression similar to the control group, confirming the absence of pro-apoptotic activity in the absence of MTX-induced injury. These findings highlight the dose-dependent anti-apoptotic potential of dulaglutide and suggest that at 0.1 mg/kg, DUL may offer greater protection against MTX-induced apoptosis than dexamethasone.

### 3.3. Effect of Dulaglutide on Fibrotic Markers

**Figure 5:**
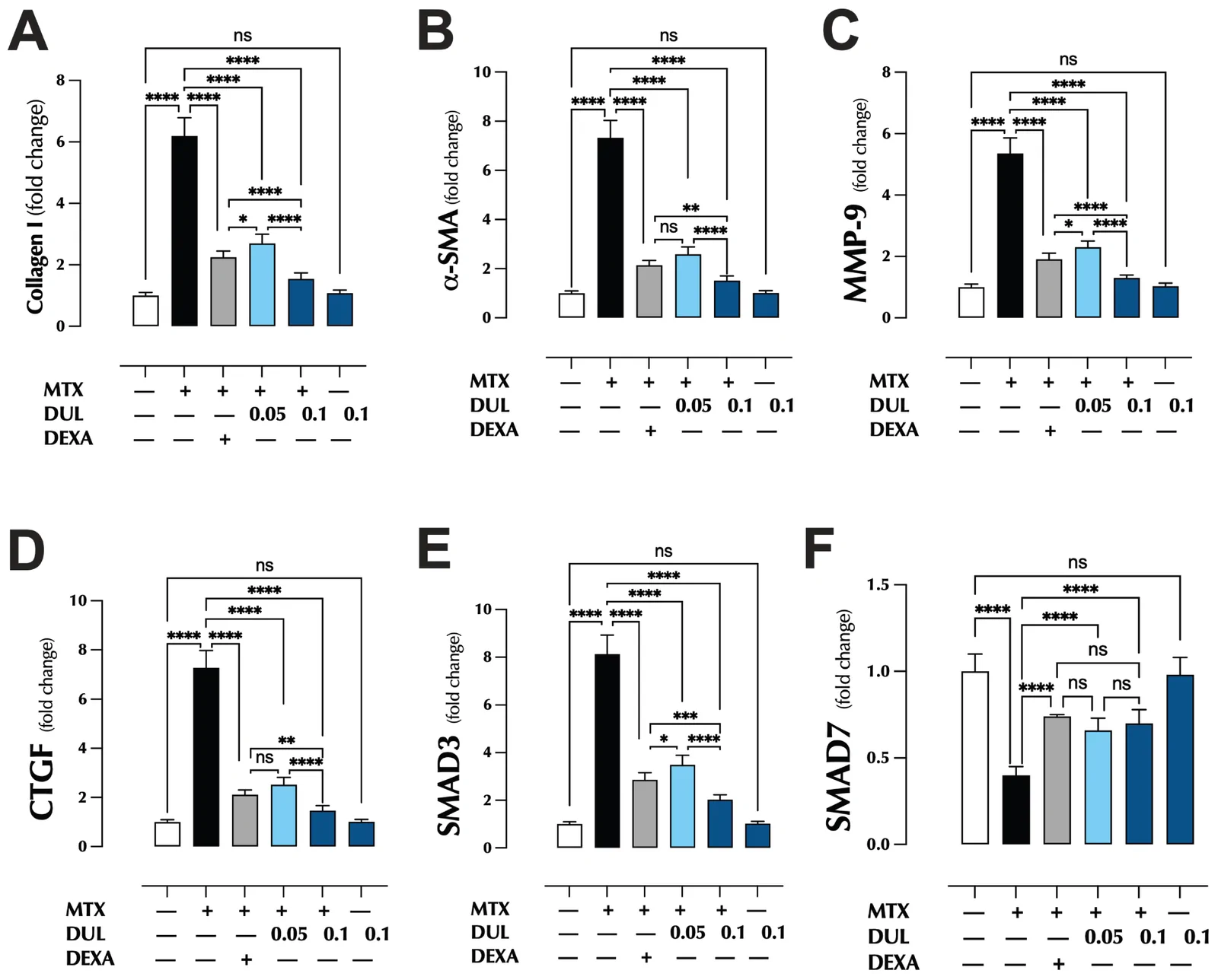
Effect of Dulaglutide on Fibrotic Markers (**A**) Collagen I, (**B**) α-SMA, (**C**) MMP-9, (**D**) CTGF, (**E**) SMAD3 and (**F**) SMAD7 in methotrexate-induced pulmonary fibrotic in rats. Values are expressed as mean ± SEM of 10 rats per group. Statistical analysis was performed by one-way ANOVA followed by Tukey’s multiple comparisons test, *p*-values were coded as follows: ns, non-significant, * *p* < 0.05, ** *p* < 0.01, *** *p* < 0.001, **** *p* < 0.0001. α-SMA; Alpha-smooth muscle actin, CTGF; Connective Tissue Growth Factor, DEXA; dexamethasone, DUL; Dulaglutide, MMP-9; Matrix Metalloproteinase-9 MTX; methotrexate. SMAD3; Mothers against decapentaplegic homolog 3 SMAD7; Mothers against decapentaplegic homolog 7.

As shown in Figure 5, gene expression analysis revealed significant alterations in key fibrotic and signaling markers in lung tissues following methotrexate (MTX) administration. Compared to the control group, MTX treatment significantly upregulated the expression of Collagen I, α-SMA, MMP-9, CTGF, and SMAD3, while markedly downregulating SMAD7 levels (*p* ≤ 0.05), indicating the activation of fibrotic and pro-apoptotic pathways. Co-treatment with DEXA, DUL 0.05 mg/kg, and DUL 0.1 mg/kg effectively mitigated these changes. Specifically, Collagen I expression was reduced by 63.6%, 56.3%, and 75.1%, respectively, compared to the MTX group. A similar trend was observed for α-SMA, which decreased by 70.8%, 64.6%, and 79.3%, respectively. MMP-9 levels were also significantly lowered by 64.3% (DEXA), 57.0% (DUL 0.05), and 75.7% (DUL 0.1), while CTGF expression was reduced by 70.9%, 65.3%, and 79.7%, respectively. SMAD3 levels decreased by 64.8%, 57.0%, and 75.0%, whereas SMAD7 expression increased by 85.0%, 65.0%, and 75.0%, respectively, in comparison to the MTX group. Notably, DUL at 0.1 mg/kg consistently demonstrated superior efficacy over both DEXA and the lower DUL dose across all markers, suggesting a dose-dependent antifibrotic and anti-apoptotic effect that may surpass the protective capacity of dexamethasone.

### 3.4. Effect on Oxidative Stress Marker

As presented in Table 2, oral administration of methotrexate (MTX) once weekly for 14 days led to a significant increase in malondialdehyde (MDA) levels, alongside a marked reduction in antioxidant markers including glutathione (GSH), catalase (CAT), superoxide dismutase (SOD), and nuclear factor erythroid 2–related factor 2 (Nrf2) in lung tissue compared to the control group (*p* ≤ 0.05). Co-treatment with DEXA, DUL 0.05 mg/kg, and DUL 0.1 mg/kg effectively counteracted these oxidative alterations. Specifically, MDA levels were reduced by 73.3%, 40.8%, and 66.5%, respectively, relative to the MTX group, with DEXA showing the strongest effect. However, when evaluating antioxidant restoration, DEXA consistently demonstrated the highest efficacy across all parameters. GSH levels increased by 310% (DEXA), 144% (DUL 0.05), and 227% (DUL 0.1), while CAT activity rose by 361%, 170%, and 270%, respectively. Similarly, SOD levels were elevated by 350%, 162%, and 202%, and Nrf2 expression increased by 396%, 175%, and 276%, respectively, compared to the MTX group. These findings suggest that while DEXA is the most potent agent in both mitigating lipid peroxidation (MDA) and upregulating antioxidant defenses, dulaglutide—particularly at the 0.1 mg/kg dose—also exerts a substantial, dose-dependent protective effect against oxidative stress in MTX-induced pulmonary injury.

### 3.5. Effect on Inflammatory Markers

**Figure 6:**
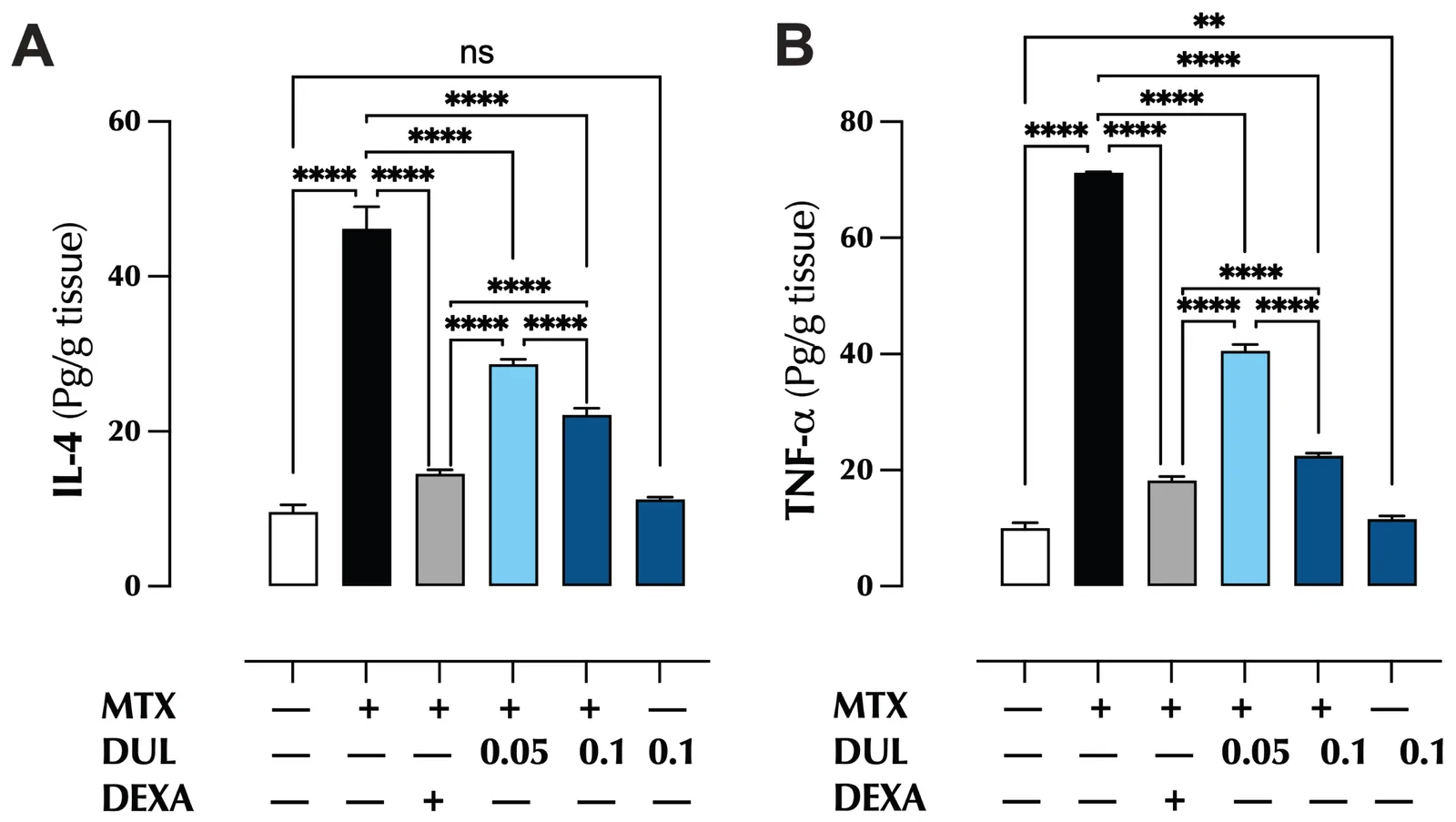
Effect of Dulaglutide on Inflammatory Markers (IL-4 (**A**) and TNF–α (**B**)) in methotrexate-pulmonary fibrotic rat. Values are expressed as mean ± SEM of 10 rats per group. Statistical analysis was performed by one-way ANOVA followed by Tukey’s multiple comparisons test, *p*-values were coded as follows: ns, non-significant, ** *p* < 0.01, **** *p* < 0.0001. MTX; methotrexate, DEXA; dexamethasone, DUL; Dulaglutide, IL-4; interleukin-4, TNF–α; Tumor Necrosis Factor-alpha.

As illustrated in Figure 6, methotrexate (MTX) administration significantly elevated the levels of pro-inflammatory cytokines TNF-α and IL-4 in rat lung tissue compared to the control group (*p* ≤ 0.05), indicating a strong inflammatory response. Co-treatment with DEXA, DUL 0.05 mg/kg, and DUL 0.1 mg/kg effectively counteracted these effects. TNF-α levels were reduced by 74.3%, 42.9%, and 68.3%, respectively, relative to the MTX group, with DEXA showing the most pronounced suppression. Similarly, IL-4 levels decreased by 68.4%, 37.8%, and 52.0%, respectively. While DEXA demonstrated stronger anti-inflammatory effects on both markers, DUL at 0.1 mg/kg showed substantial efficacy, outperforming the lower DUL dose and approaching the effectiveness of DEXA. These findings suggest that dulaglutide, particularly at higher doses, offers a promising anti-inflammatory profile in mitigating MTX-induced pulmonary inflammation.

### 3.6. Effect on Apoptosis Markers

**Figure 7:**
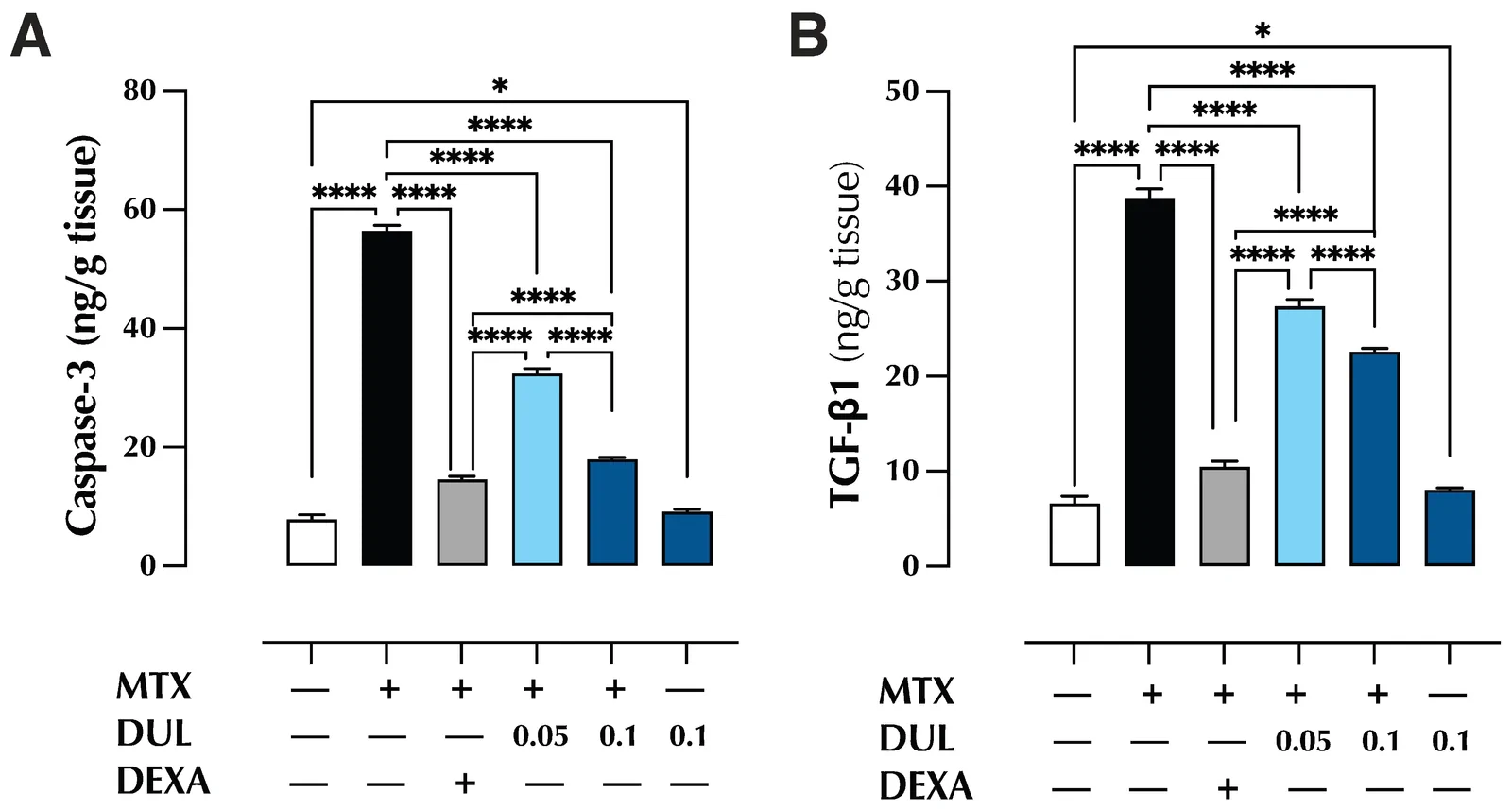
Effect of Dulaglutide on CASPASE-3 (**A**) and TGF-β1 (**B**) Expression in MTX-Induced Lung Fibrosis in rats. Values are expressed as mean ± SEM of 10 rats per group. DEXA; dexamethasone, DUL; Dulaglutide, MTX; methotrexate, TGF-β1; Transforming growth factor beta 1. Statistical analysis was performed by one-way ANOVA followed by Tukey’s multiple comparisons test, *p*-values were coded as follows: * *p* < 0.05, **** *p* < 0.0001.

As shown in Figure 7, methotrexate (MTX) administration significantly increased CASPASE-3 and TGF-β1 levels in lung tissue compared to the control group (*p* ≤ 0.05), indicating enhanced apoptotic and profibrotic activity. Co-treatment with DEXA, DUL 0.05 mg/kg, and DUL 0.1 mg/kg markedly reduced these elevations. CASPASE-3 expression decreased by 74.2%, 42.5%, and 68.1%, respectively, relative to the MTX group, with DEXA showing the strongest anti-apoptotic effect, followed closely by DUL at 0.1 mg/kg. Similarly, TGF-β1 levels were reduced by 72.9% (DEXA), 29.2% (DUL 0.05), and 41.5% (DUL 0.1). These findings suggest that while DEXA provides superior suppression of both apoptosis and fibrosis markers, DUL at the higher dose demonstrates considerable efficacy, outperforming the lower dose and offering a promising alternative for mitigating MTX-induced lung injury.

### 3.7. Effect of Dulaglutide on AMPK Activation in Methotrexate-Induced Pulmonary Fibrosis

**Figure 8:**
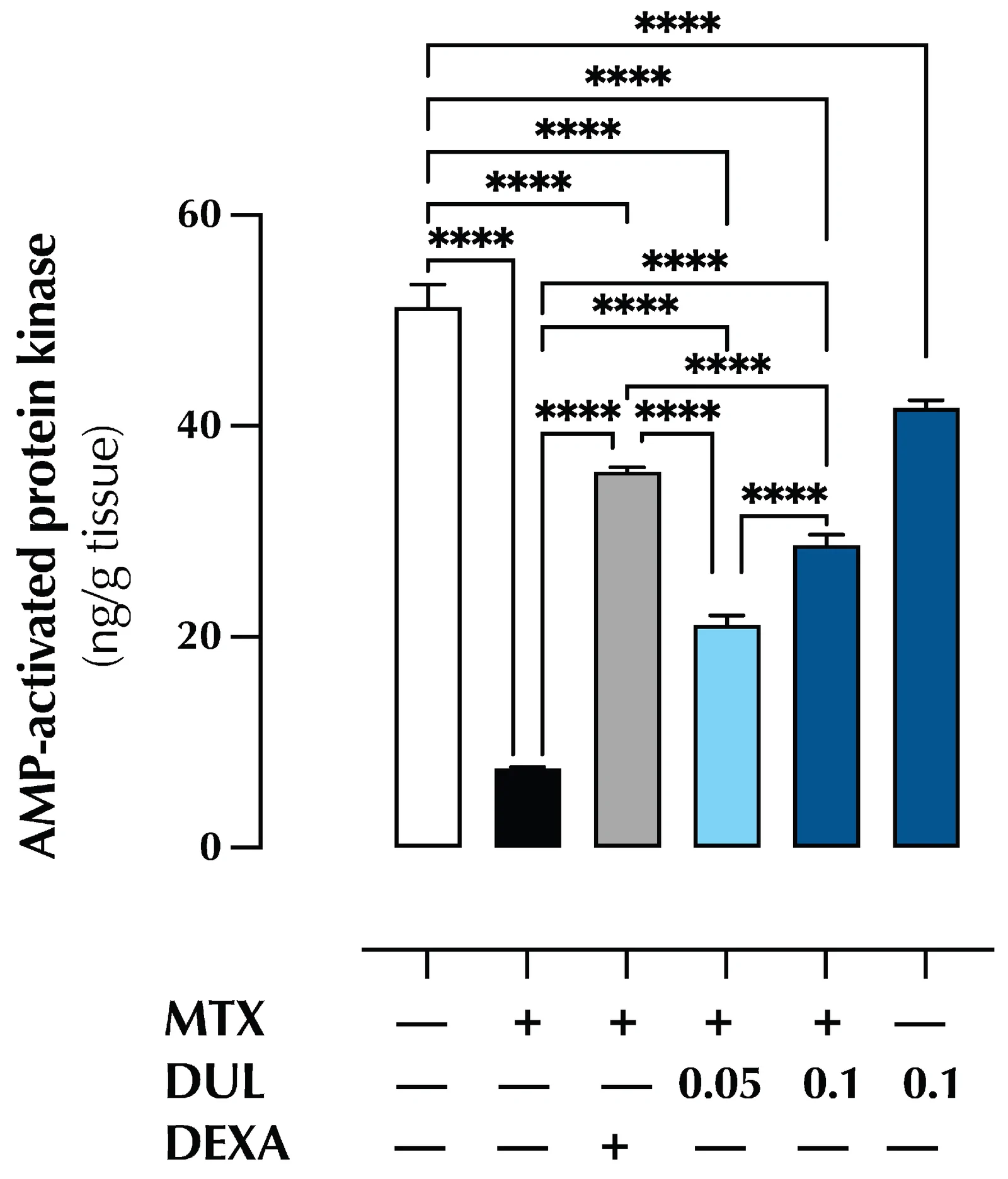
Effect of Dulaglutide on AMPK Activation in MTX-Induced Lung Fibrosis in rats. Values are expressed as mean ± SEM of 10 rats per group. Statistical analysis was performed by one-way ANOVA followed by Tukey’s multiple comparisons test, *p*-values were coded as follows: **** *p* < 0.0001. DEXA; dexamethasone, DUL; Dulaglutide, MTX; methotrexate.

As shown in Figure 8, methotrexate (MTX) administration significantly reduced AMPK levels in lung tissue compared to the control group (*p* ≤ 0.05), indicating impaired cellular energy regulation. Co-treatment with DEXA, DUL 0.05 mg/kg, and DUL 0.1 mg/kg markedly restored AMPK expression by 375%, 198%, and 301%, respectively, relative to the MTX group. While DEXA exhibited the strongest activation of AMPK, DUL at 0.1 mg/kg demonstrated substantial improvement, outperforming the lower DUL dose and approaching the effect of DEXA. These findings suggest that dulaglutide enhances AMPK signaling in a dose-dependent manner, contributing to its protective role against MTX-induced pulmonary injury.

### 3.8. Effect of Dulaglutide on Beclin-1 Expression in MTX-Induced Pulmonary Injury

**Figure 9:**
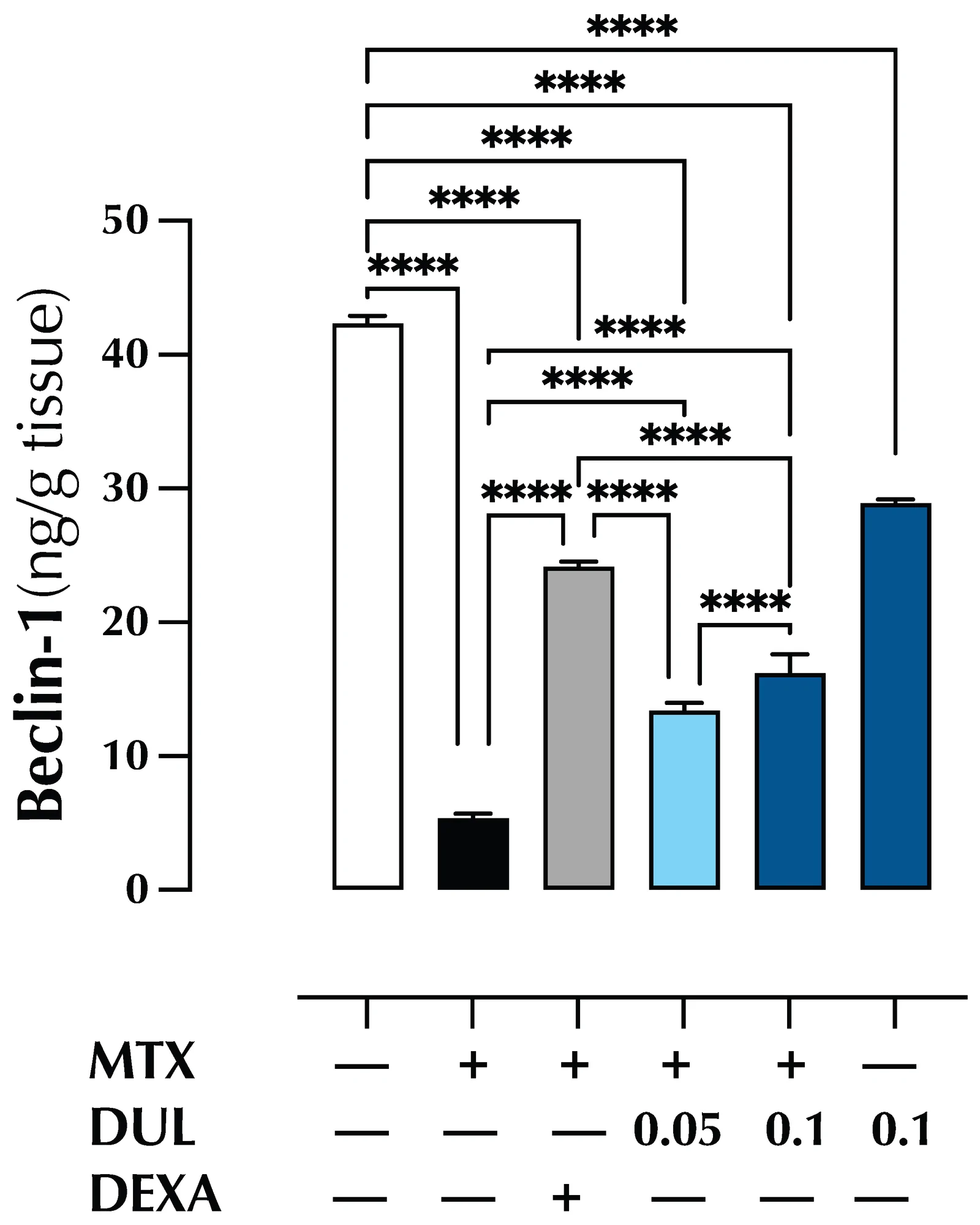
Effect of Dulaglutide on Beclin-1 Expression in MTX-Induced Lung Fibrosis in rats. Values are expressed as mean ± SEM of 10 rats per group. Statistical analysis was performed by one-way ANOVA followed by Tukey’s multiple comparisons test, *p*-values were coded as follows: **** *p* < 0.0001. DEXA; dexamethasone, DUL; Dulaglutide, MTX; methotrexate.

As illustrated in Figure 9, oral administration of methotrexate (MTX) once weekly for 14 days resulted in a significant reduction in the autophagy marker Beclin-1 within lung tissue compared to the control group (*p* ≤ 0.05). Conversely, co-treatment with dexamethasone (DEXA), dulaglutide (DUL) at 0.05 mg/kg, and DUL at 0.1 mg/kg markedly increased Beclin-1 expression by 335%, 168%, and 242%, respectively, relative to the MTX group. Among these interventions, DEXA elicited the most pronounced effect, while the higher DUL dose demonstrated superior efficacy compared to the lower dose, indicating a dose-dependent response.

## 4. Discussion

The investigation’s findings clearly demonstrate that MTX induces pulmonary fibrosis in rats, as evidenced by elevated levels of oxidative, inflammatory, and apoptotic markers in lung tissue. Key findings from the study suggest that MTX triggers oxidative damage, inflammation, and apoptosis, in lung and blood tissue through distinct biomolecular pathways. Peripheral fibrosis is believed to result from inflammatory cells infiltration into the lung which releasing mediators that interact with resident lung cells, subsequently stimulating collagen deposition and fibroblast proliferation in the lung interstitium [31]. Furthermore, the apoptosis of parenchymal cells specifically in the alveolar epithelium and capillary endothelial cells has been shown to facilitate collagen accumulation and the expansion of fibroblast and myofibroblast populations [32]. Among the known mechanisms of MTX-induced tissue damage, reactive oxygen species (ROS) generation is considered the most prominent [33].

Histological analysis revealed a marked increase in fibrosis following MTX administration compared to control animals. However, treatment with of DUL at both 0.05 mg/kg/week and 0.1 mg/kg/week significantly reduced fibrosis levels, as confirmed by hematoxylin and eosin (H&E) and Masson’s trichrome (MTC) staining. Supporting previous reports, MTX was found to damage alveolar epithelial cells, compromising the integrity of the alveolar-capillary basement membrane and promoting subsequent collagen deposition along with the recruitment and proliferation of myofibroblasts [34]. Consistent with our results, other studies have highlighted key histopathological alterations in MTX-treated lungs, including congestion, inflammatory cell infiltration, hemorrhage, and cellular degeneration [35]. Moreover, excessive ROS generation mediated via MTX can induce the translocation of Bax to the outer mitochondrial membrane, facilitating cytochrome-c release, leading to activation of the caspase cascade, ultimately resulting in apoptosis [36].

The results showed that the levels of Collagen I, α-SMA, MMP-9, CTGF, SMAD3 and SMAD7 levels were significantly elevated in the lung of rats treated with methotrexate (14 mg/kg/week) compared to the control group. Treatment with dulaglutide (DUL) at both 0.05 mg/kg/week and 0.1 mg/kg/week significantly reduced the expression of Collagen I, α-SMA, MMP-9, CTGF, and SMAD3, while notably increasing SMAD7 expression in a dose-dependent manner compared to the MTX group. Additionally, the current study showed a significant increase in TGF-β1 concentration following MTX administration. TGF-β1 is a key cytokine involved in several physiological and pathological processes, including wound healing, immunosuppression, fibrosis, and cell proliferation. It functions as immunosuppressive mediator and regulates cellular growth and division [37]. Under normal conditions, TGF-β binds to its receptor, TGF-βRIIβ, which subsequently activates the TGF-βRI kinase leading to phosphorylation of receptor-regulated Smads (Smad3) [38]. Phosphorylated Smad3 translocate to the nucleus, where it regulates the transcription of target genes, including α-SMA and those involved in extracellular matrix (ECM) production [39]. The observed increase in TGF-β1 expression closely correlates with elevated α-SMA levels. This relationship is likely due to TGF-β’s role in activating resident fibroblasts, promoting their differentiation into myofibroblasts that strongly express α-SMA [40]. Moreover, TGF-β1 also influences ECM remodeling by regulating the expression of matrix metalloproteinases (MMPs) and their tissue inhibitors (TIMPs) [41].

GLP-1 receptor agonists, including dulaglutide, have been reported to attenuate ROS buildup and endothelial cells’ oxidative stress-induced cell dysfunction via GLP-1-dependent signaling pathways [42,43,44]. During the fibrotic phase, GLP-1 therapy demonstrated efficacy in reducing collagen synthesis and deposition. Likewise, GLP-1 receptor activation has been shown to alleviate fibrosis in various models of cardiac and renal disease [45]. Overexpression of Smad7 has been associated with reduced fibrogenic responses, partly by suppressing intracellular ROS production and potentially lowering MMP-9 activity [46,47]. GLP-1 also inhibits Smad3 activation, thereby preventing epithelial–mesenchymal transition (EMT) and ECM deposition [48]. Furthermore, our findings suggest that GLP-1 reduces connective tissue growth factor (CTGF) expression in the early phases of the inflammatory response [49]. In alignment with previous studies, our data also showed that GLP-1 significantly downregulated α-SMA expression in a murine model of bleomycin-induced lung fibrosis [47].

Moreover, the results showed that the levels of GSH, CAT and SOD levels were significantly decreased, while significantly increased in the lungs of rats treated with MTX (14 mg/kg/week) compared to the control group. In contrast, treatment with dulaglutide (DUL) at doses of 0.05 mg/kg/week and 0.1 mg/kg/week significantly increased GSH, CAT and SOD levels and significantly reduced MDA levels in a dose-dependent manner. These findings indicate that oxidative stress contributes to MTX-induced lung injury, likely due to an imbalance between reactive oxygen species (ROS) production and antioxidant defense mechanisms [50,51]. SOD is a metal-containing enzyme primarily located in the mitochondria, where it catalyzes the dismutation of superoxide radicals into hydrogen peroxide (H_2_O_2_). Glutathione peroxidase (GPx) further converts H_2_O_2_ into water by oxidizing GSH, while catalase breaks down H_2_O_2_ into water and oxygen, preventing its conversion into highly toxic hydroxyl radicals [52]. In contrast to MTX-induced oxidative damage, treatment restored antioxidant defense by upregulating key enzymes such as GSH, SOD, and CAT. Notably, glutathione, a vital ROS scavenger, is often deficient in the alveolar lining fluid of individuals with pulmonary oxidative injury [53].

Moreover, MTX treatment resulted in a significant reduction in nuclear factor erythroid 2–related factor 2 (Nrf2) levels in lung tissue compared to controls. DUL administration at both tested doses significantly upregulated Nrf2 expression in a dose-dependent manner. Nrf2 is a critical transcription factor regulating the cellular antioxidant response. Previous in vitro and in vivo studies have demonstrated that GLP-1 receptor agonists activate the Nrf2 pathway, thereby reducing ROS levels and enhancing the expression of antioxidant enzymes [54].

Inflammatory cytokines were also markedly affected. MTX-treated rats exhibited a significant increase in tumor necrosis factor-alpha (TNF-α) and interleukin-4 (IL-4) levels in lung tissue compared to controls. However, DUL treatment at 0.05 and 0.1 mg/kg/week significantly reduced TNF-α and IL-4 levels in a dose-dependent manner. Previous studies have confirmed that inflammation plays a central role in the development of fibrosis [55]. MTX increases TNF-α [56], and IL-4 Levels [57], which are key pro-inflammatory cytokines involved in promoting inflammatory responses and enhancing oxidative stress via nitric oxide (NO) and other mediators [58]. On other hand, GLP-1 receptor agonists not only reduce the amounts of TNF-α, IL-4, and total inflammatory cell count in bronchoalveolar lavage fluid in experimental models of lung inflammation [59].

Consequently, MTX treatment raises TGF beta1 and Caspase-3 levels in lung tissue compared to the control group. In contrast, treatment with dulaglutide (DUL) at 0.05 mg/kg/week and 0.1 mg/kg/week significantly reduced both TGF-β1 and Caspase-3 levels in a dose-dependent manner. TGF-β1 is expressed in alveolar macrophages during the early phase of inflammatory cell infiltration, and in epithelial cells during the later phase of PF [60]. Prior studies have suggested that GLP-1 receptor agonists may mediate reductions in TGF-β1 and caspase-3 levels, thereby limiting alveolar epithelial cell apoptosis in PF. This effect is thought to be driven by a reduction in reactive oxygen species (ROS) produced by inflammatory cells (e.g., neutrophils and macrophages) and parenchymal cells such as myofibroblasts, which otherwise contribute to epithelial cell death [61].

In the present study, MTX administration (14 mg/kg/week) induced a significant decrease in AMPK level in lung tissue as compared to control. However, DUL therapy at both 0.05 mg/kg/week and 0.1 mg/kg/week markedly increased AMPK expression levels in a dose-dependent manner relative to the MTX group. This reduction in AMPK by MTX is consistent with earlier research in liver tissue, which demonstrated that methotrexate metabolites inhibit DNA synthesis and suppress AMPK activity [62]. GLP-1 inhibits vascular smooth muscle cell proliferation by activating AMPK [63], and pharmacological activation of AMPK has been associated with reduced oxidative stress, inflammation, and apoptosis, leading to improved organ structure and function [64]. AMPK activation also promotes the expression of antioxidant enzymes, thereby limiting ROS generation [65].

Additionally, the study found that MTX significantly decreased Beclin-1 levels in lung tissue compared to the control group (*p* < 0.05). Treatment with DUL at 0.05 and 0.1 mg/kg/week significantly increased Beclin-1 levels in a dose-dependent manner compared to the MTX group. Beclin-1 is a key regulator of autophagy, a vital cellular defense mechanism influenced by oxidative stress and ROS. Autophagy facilitates the degradation of intracellular pathogens and damaged cellular components through lysosomal pathways [66]. Our results show that MTX significantly suppresses Beclin-1 levels in lung tissues, suggesting a shutdown of protective autophagic pathways. Dulaglutide treatment successfully restored Beclin-1 expression, pointing to a recovery of cytoprotective cellular clearance. This aligns with previous findings where GLP-1 receptor agonists upregulated Beclin-1 and restored cytoprotective autophagy to rescue cells from severe oxidative stress and drug-induced tissue damage [67,68].

Overall, dexamethasone was more effective in reducing lipid peroxidation and suppressing inflammatory cytokines, while DUL demonstrated superior efficacy in restoring antioxidant defenses, reducing fibrosis, enhancing autophagy. Given the limitations of corticosteroid therapy, including immunosuppression and metabolic side effects, the ability of DUL to target multiple pathogenic pathways—oxidative stress, apoptosis, fibrosis, and energy dysregulation—positions it as a novel therapeutic candidate. The dose-dependent benefits of DUL, particularly at 0.1 mg/kg, highlight its potential as a promising alternative or adjunct to corticosteroids for managing MTX-induced pulmonary toxicity.

## 5. Conclusions

In conclusion, the current study showed that MTX induces significant oxidative stress, inflammation, and apoptosis in mesenchymal and epithelial lung cells. MTX treatment led to marked pulmonary damage, including hyperemia, tissue deterioration. These pathological effects were substantially mitigated by dulaglutide treatment, which restored antioxidant capacity, reduced inflammatory and fibrotic markers, and improved lung histology in a dose-dependent manner. Specifically, our findings show a strong association between dulaglutide’s protective effects and the upregulation of total AMPK and Beclin-1 levels to restore cytoprotective autophagy, while suppressing TGF-β1/Smad signaling to prevent myofibroblast activation and collagen deposition.

In terms of practical applications, these results position dulaglutide—an established GLP-1 receptor agonist—as a promising therapeutic candidate or adjunct therapy to mitigate methotrexate-induced pulmonary toxicity, potentially offering a safer alternative to long-term corticosteroid use. Future research directions should focus on validating these mechanisms using AMPK- or Beclin-1-knockout models to confirm direct causality, evaluating the combination of dulaglutide with low-dose corticosteroids, and initiating clinical studies to assess the translational safety and efficacy of GLP-1 receptor agonists in patients undergoing methotrexate therapy.

## 6. Limitations

Clinical MTX pulmonary toxicity presents as acute pneumonitis or chronic fibrosis over years of therapy. While our accelerated model (14 mg/kg/week, p.o., for 2 weeks) cannot match the clinical timeline, it successfully reproduces hallmark pathological features, including acute alveolar damage, dense inflammatory cell infiltration, and rapid collagen deposition, serving as a reliable platform for evaluating early-stage interventions. While our study demonstrates an association between AMPK/Beclin-1 pathway restoration and reduced lung injury, further studies using genetic knockouts or specific inhibitors (e.g., AMPK or Beclin-1 inhibitors) are required to definitively establish direct causality.

## Figures and Tables

**Table 1:** GenBank accession numbers, primer sequences and annealing temperatures of the assessed genes.

GenBank Accession No.	GenBank	Primers	Annealing Temperature
NM_053304	Collagen-1	Forward: 5′-CATGACCGAGACGTGTGGAA-3′ Reverse: 5′-GCAGGCGAGATGGCTTATTTG-3′	53 °C
NM_031004	α-SMA	Forward: 5′-CCGACCGAATGCAGAAGGA-3′ Reverse: 5′-ACAGAGTACTTGCGCTCTGA-3′	50 °C
NM_031055	MMP-9	Forward: 5′-TGTACCGCTATGGTTACACTCG-3′ Reverse: 5′-GGCAGGGACAGTTGCTTCT-3′	53 °C
NM_022266	CTGF	Forward: 5′-CAATACCTTCTGCAGGCTGGA-3′ Reverse: 5′-TTAGCCCGGTAGGTCTTCACA-3′	52 °C
NM_013095	SMAD3	Forward: 5′-CATGGGCAAATGAAAGGGCT-3′ Reverse: 5′-CCAGGGTGAAGATGACAGGT-3′	55 °C
NM_001042660.1	SMAD7	Forward: 5′-GGGTTTACAACCGCAGCAGT-3′ Reverse: 5′-GCCTTGATGGAGAAACCAGG-3′	53 °C
NM_031144	β-actin	Forward: 5′-CACCCGCGAGTACAACCTTC-3′ Reverse: 5′-CCCATACCCACCATCACACC-3′	53 °C

**Table 2:** Effect of Dulaglutide on Oxidative Stress Markers (MDA, GSH, CAT, SOD and Nrf2) in methotrexate-pulmonary fibrotic rats.

Group	MDA (nmol/g Tissue)	GSH (Pg/g Tissue)	CAT (U/g Tissue)	SOD (U/g Tissue)	Nrf2 (Pg/g Tissue)
Control	11.99 ± 2.05	49.85 ± 2.76	62.16 ± 2.9	52.01 ± 3.32	41.15 ± 3.18
Methotrexate (14 mg/kg)	80.59 ± 7.42 a	8.32 ±1.34 a	8.90 ± 1.63 a	6.483 ± 1.41 a	6.10 ±0.92 a
Methotrexate+ Dexamethasone (0.5 mg/kg)	21.48 ± 1.56 b	34.18 ± 0.78 b	41.10 ± 1.41 b	29.20 ± 0.57 b	28.65 ± 0.35 b
Methotrexate+ Dulaglutide (0.05mg/kg)	47.66 ± 1.06 b, c	20.34 ± 1.34 b, c	24.10 ± 1.84 b, c	16.99 ± 0.85 b, c	16.8 ±1.27 b, c
Methotrexate+ Dulaglutide (0.1 mg/kg)	26.99 ± 0.99 b, d	27.23 ± 1.91 b, c, d	32.98 ± 2.55 b, c, d	19.63 ± 2.97 b, c	22.95 ± 1.91 b, c, d
Dulaglutide (0.1 mg/kg)	13.54 ± 1.2	40.24 ± 0.78	48.75 ± 1.63	35.39 ± 1.13	33.6 ± 1.13

Values are expressed as mean ± SEM of 10 rats per group. a vs. control group, b vs. MTX group, c vs. MTX + DEXA, d vs. MTX + DUL 0.05 (one-way ANOVA followed by Tukey multiple comparisons test; *p* < 0.05). CAT; catalase, GSH; reduced glutathione, MDA; Malondialdehyde, Nrf2; Nuclear factor erythroid 2, SOD; Superoxide Dismutase.

## Data Availability

The original contributions presented in this study are included in the article. Further inquiries can be directed to the corresponding author.
